# Exploring the Occupational Lifestyle Experiences of the Families of Public Safety Personnel

**DOI:** 10.1007/s10926-024-10179-x

**Published:** 2024-03-28

**Authors:** Rachel Richmond, Rosemary Ricciardelli, Rachel Dekel, Deborah Norris, Alyson Mahar, Joy MacDermid, Nicola T. Fear, Rachael Gribble, Heidi Cramm

**Affiliations:** 1 Queen’s University, Kingston, Canada; 2 Memorial University of Newfoundland, St. John’s, Canada; 3 Bar Ilan University, Ramat Gan, Israel; 4 Mount Saint Vincent University, Halifax, Canada; 5 University of Western Ontario, London, Canada; 6 Academic Department of Military Mental Health, King’s College London, London, UK; 7 King’s Centre for Military Health Research, King’s College London, London, UK

**Keywords:** Public safety personnel, Family perspective, Qualitative, Systematic review, Occupational rehabilitation

## Abstract

**Purpose:**

Public safety personnel, including first responders, are regularly exposed to physical, social, and psychological risks and occupational requirements. These risks and requirements extend beyond the employee and may also impact the families (for example, work-family conflict, compassion fatigue). Despite recent attention directed at the population’s wellness, considerably less attention is directed towards the family. This review investigates how the risks and requirements associated with these occupations affect families’ lives and experiences, and correspondingly, how families respond and adapt to these risks.

**Methods:**

In the current qualitative review, we sought to identify and describe the lifestyle experiences of public safety families as they navigate the occupational risks and requirements of public safety work. The inclusion criteria resulted in an analysis of 18 articles, representing only police (*n* = 11), paramedics (*n* = 7), and firefighting (*n* = 10) sectors.

**Results:**

We identified and described the experiences of public safety families both by occupation and familial role. Shared familial themes across occupational groups included ‘Worry’, ‘Communication’, ‘Where do I turn’, ‘Are they okay’, ‘Serving alongside’, and ‘(Over)Protective’. However, distinct themes also emerged between different occupational groups and family configurations. Themes prevalent amongst primarily children of police included: ‘Worry’, ‘Let’s Laugh Instead’, ‘(Over)Protective’, and ‘I’m not the Police, my Parent is!’. Experiences differed if the family contained on serving public safety personnel or multiple.

**Conclusion:**

We identified the shared and unique occupational experiences of public safety families. This review normalizes these experiences and emphasizes the need to develop initiatives to improve the well-being of families and safety employees.

**Supplementary Information:**

The online version contains supplementary material available at 10.1007/s10926-024-10179-x.

## Background

Public safety personnel (PSP), including but not limited to firefighters, paramedics, police, correctional workers, and public safety communicators [1], are routinely exposed to numerous risks to their mental well-being such as potentially psychologically traumatic events, insomnia, and risks of physical injury or death [1, 2]. In a Canadian study, 44.5% of PSP screened positive for symptoms consistent with one or more mental health disorders, including major depressive disorder (26.4%) and posttraumatic stress disorder (23.2%). Moreover, 27.8% reported lifetime suicidal ideation [3]. These states persist and can also impact families, where over 70% of PSP report seeking support from partners as early resources when in compromised states of wellness or crisis [4].

While partners generally feel proud and support the work their loved ones do for the community [5, 6], partners also report negative impacts associated with the public safety occupations, such as increased familial stress, worrying about their partner’s safety, relationship strain, anger, compassion fatigue, marital breakdowns, feeling like a single parent, and the need to anticipate and manage their partner’s moods [5–8]. Additionally, many public safety families experience these impacts in isolation, often internalizing these consequences as their own fault, rather than the outcomes of public safety work [9]. The stress from public safety work transferring into the home can be the impetus for work-family conflict leading to partner overload, partner distress, vicarious trauma, and/or family crisis [10–12]. When there is elevated work-family conflict, employers have noticed increased absenteeism, turnover, and low recruitment [13–15]. Organizations have begun to provide family-based initiatives in an effort to increase recruitment & retention, performance & productivity, and organizational culture & job satisfaction [16]. However, these initiatives need to be tailored to the needs and requirements of the employees and families of the specific occupation to be most effective.

Little attention is paid in the qualitative literature to the familial perspective on how the risks and requirements associated with public safety occupations impact family life, and correspondingly, how families respond and adapt to the impacts both relating to the familial unit themselves and the operationalization of the PSP. In order to properly develop and implement effective family-based initiatives in public safety organizations, this review sought to qualitatively identify and describe the shared and unique experiences of PSP families as they navigate through the occupational risks and requirements of working in public safety across occupational groups. A secondary purpose of this review is to normalize the experiences of public safety families to decrease the stigma and isolation associated with the occupational risk and requirements of public safety occupations.

## Methods

To understand the experiences of public safety families from their own perspectives, we conducted a qualitative systematic review adhering to the Joanna Briggs Institute (JBI) methodology for systematic reviews of qualitative evidence [17, 18]. Six databases (Embase, MEDLINE, Web of Sciences, CINAHL, PsycINFO, and Sociological Abstracts) were searched in June 2021 with the consultation of librarians to identify qualitative studies whose authors included an examination of the experiences of PSP family members. Utilizing the expertise of the librarians we conducted our search in order to capture an array of peer-reviewed literature to ensure our desired data were captured. Our search strategy included three categories of terms: overarching occupational terms (e.g. public safety personnel, PSP, emergency responder, first responder), specific occupational terms (e.g. border service officers, public safety communicators, correctional officers, firefighters, Indigenous emergency managers, operational intelligence personnel, paramedics, police, search and rescue), and familial terms (e.g. family, spouse, partner, significant other, children, parent), with the appropriate Boolean operators. For a more exhaustive list, please see online Appendix A. In addition to our wide search, our review did not implement any temporal or geographical restrictions. We did limit our analyses to documents that were available in their entirety in English due to the language limitations of the authors. The search was updated in October 2022 to ensure timeliness.

All returns were entered into Covidence [19] where duplicates were removed. In accordance with the JBI methodology for systematic reviews of qualitative evidence [17], two screeners independently screened each document by title and abstract. This phase included all abstracts relating to families of PSP, from any perspective, and utilizing any methodology. Articles were then screened by their full texts, utilizing selection criteria restricting our analysis to include only articles from the familial perspective, about loved ones of public safety personnel from firefighters, police, paramedics, communications, and/or correctional sectors, and articles that employed a qualitative method in at least one section of the analysis. As this review aimed to capture the normal lifestyle experiences of public safety families, articles relating solely to specific disasters such as 9/11 were also excluded. Any differences of opinions regarding article inclusion were discussed and resolved amongst the research team, where a unanimous vote ensured inclusion.

Data were extracted by segments—the written text was divided by meaningful units and ideas—to ensure quotes were kept in their entireties. Data were evaluated for quality, adhering to the principles of the JBI methodology for systematic reviews of qualitative evidence, utilizing their three categories: unequivocal, credible, and unsupported [17]. Segments of articles categorized as unequivocal had quotes with clear meanings beyond reasonable doubt. Segments categorized as credible included quotes whose meanings lacked clarity, and segments where the author makes a statement unaccompanied by data were categorized as unsupported [17]. Data extraction was conducted in three phases. First, one article was selected at random, and RR, HC, and RD extracted segments and categorized them together by quality to establish a baseline of understanding. Second, three additional articles were selected at random, and RR, HC, and RD all extracted the segments by quality separately, establishing calibration across articles and authors. Finally, RR completed the remaining articles with regular discussions with HC and RD. This process ensured the rigour of the extraction process. To maintain rigour in our overall review, only unequivocal data were kept for analysis.

In continuing with the JBI methodology for systematic reviews of qualitative evidence, a meta-aggregation approach was used to synthesize and analyse the data both by PSP sector, and then familial role [20]. This approach avoids re-interpretation of the included studies, but rather, ensures an accurate and reliable representation of those findings to ensure trustworthiness of the reporting. Once all the findings were extracted with an accompanying quote and assessed as an unequivocal finding, these findings were paired with others of a similar nature to form categories. These categories are then grouped together based on similarity until they form themes. This process is iterative and was conducted with consultation of all the authors, until consensus was reached.

## Results

The search yielded 19,107 results, comprising of 17,121 original articles. Of those articles, 124 articles were screened for full text and 17 were included in the initial review. Following the search update in October 2022, an additional article was added; thus, 18 articles were included for the final analysis (see Table 1). A full PRISMA diagram can be found in online Appendix B. The 18 included articles were published between 2005 and 2022, which originated from six countries, with most studies (10) conducted in the United States of America (USA). Familial data were primarily from female heterosexual-presenting partners of male PSP, with only three articles including the voices of PSP children. Despite searching for multiple public safety sectors, qualitative data from the family were only present for police, paramedic, and firefighter sectors, with no representation from correctional or public safety communicator families. The data extraction process yielded 542 segments, each assessed for quality according to the JBI methodology for systematic reviews of qualitative evidence [17]. After the quality control process, 365 unequivocal segments were kept for analysis, while 68 credible, and 205 unsupported segments were excluded due to a lack of quality evidence. We present analysed data first by occupational group, then by familial role.

**Table 1:** Overview of Included Articles

Author and date	Country	PSP sector	# of participants	Type of participant	Sex	Qualitative method used
Alrutz et al. [33]	New Zealand	Police, Fire, Paramedic & Military	664 [458 were partners of PSP (27%-police, 33%-fire, 13%-paramedic)]	Spouse/Significant Other	89% female	Survey with open ended questions
Amendola et al. [62]	USA	Police	16–20 Spouse/Significant Other	Spouse/Significant Other	Unknown	Scale Development with Focus Groups
Bochantin [29]	USA	Police & Fire	95 (36 PSP, 59 Family Members)	PSP, Spouse/Significant Other, & children	70% female partners/spouses, 50% female children	Interviews and focus groups
Bochantin [22]	USA	Police & Fire	95 (36 PSP, 59 Family Members)	PSP, Spouse/Significant Other, & children	70% female partners/spouses, 50% female children	Interviews and focus groups
Brodie [26]	USA	Police	7 partner Diads	PSP & Spouse/Significant Other (1 dual serving)	Unknown	Interviews
Helfers et al. [32]	USA	Police	19 Children	Children	42% female	Interviews
Hill et al. [31]	United Kingdom	Fire	10 (9 Female Spouse/Significant Others, & 1 Male Sibling)	Spouse/Significant Other & a Sibling	90% female	Interviews
Karaffa et al. [23]	USA	Police	82 Officers, 89 Spouse/Significant Other	PSP & Spouse/Significant Other	95% of officers were male, 98% of spouses were female	Needs assessment with open ended questions
Landers et al.[25]	USA	Police/Corrections	8 Spouse/Significant Other	Spouse/Significant Other	100% female	Interview
Lawn et al. [27]	Australia	Police, Fire, Paramedic, Veteran	25 Family Members (2- Paramedic, 1- Fire, 15- Police)	Spouse/Significant Other, Adult Son, & a Mother	76% Female	Interviews
Porter & Henriksen [6]	USA	Police, Fire, Paramedic	6 Spouse/Significant Other (1- Paramedic, 2-Fire, 3-Police)	Spouse/Significant Other	83% female	Interviews
Regehr [24]	Canada	Paramedic	14 Spouse/Significant Other	Spouse/Significant Other	93% female	Interviews
Regehr et al. [5]	Canada	Fire	14 Spouse/Significant Other	Spouse/Significant Other	100% Female	Interviews
Roth & Moore [21]	USA	Paramedic	12 (11 Spouse/Significant Other, 1 parent)	Spouse/Significant Other & a Parent	83% female	Interviews
Sommerfeld et al. [30]	Canada	Fire	19 participants	PSP & Spouse/Significant Other	100%-male PSP, 100% female spouses	Interviews
Waddell et al. [9]	Australia	Police, Fire, Paramedic & Veteran	22 Spouse/Significant Other (5- Paramedic, 5-Fire, 2-Police)	Spouse/Significant Other	90% female	Interviews
Watkins et al. [28]	USA	Fire	48 (38 Firefighters, 4 Battalion Chiefs, 6 Spouse/Significant Other)	PSP & Spouse/Significant Other	Majority of PSP were male, Majority of partners were female	Focus groups
Wheater & Erasmus [17]	South Africa	Paramedic	8 Spouse/Significant Other	Spouse/Significant Other	63% female	Interviews

### Family Experiences by Occupational Group

**Fig. 1:**
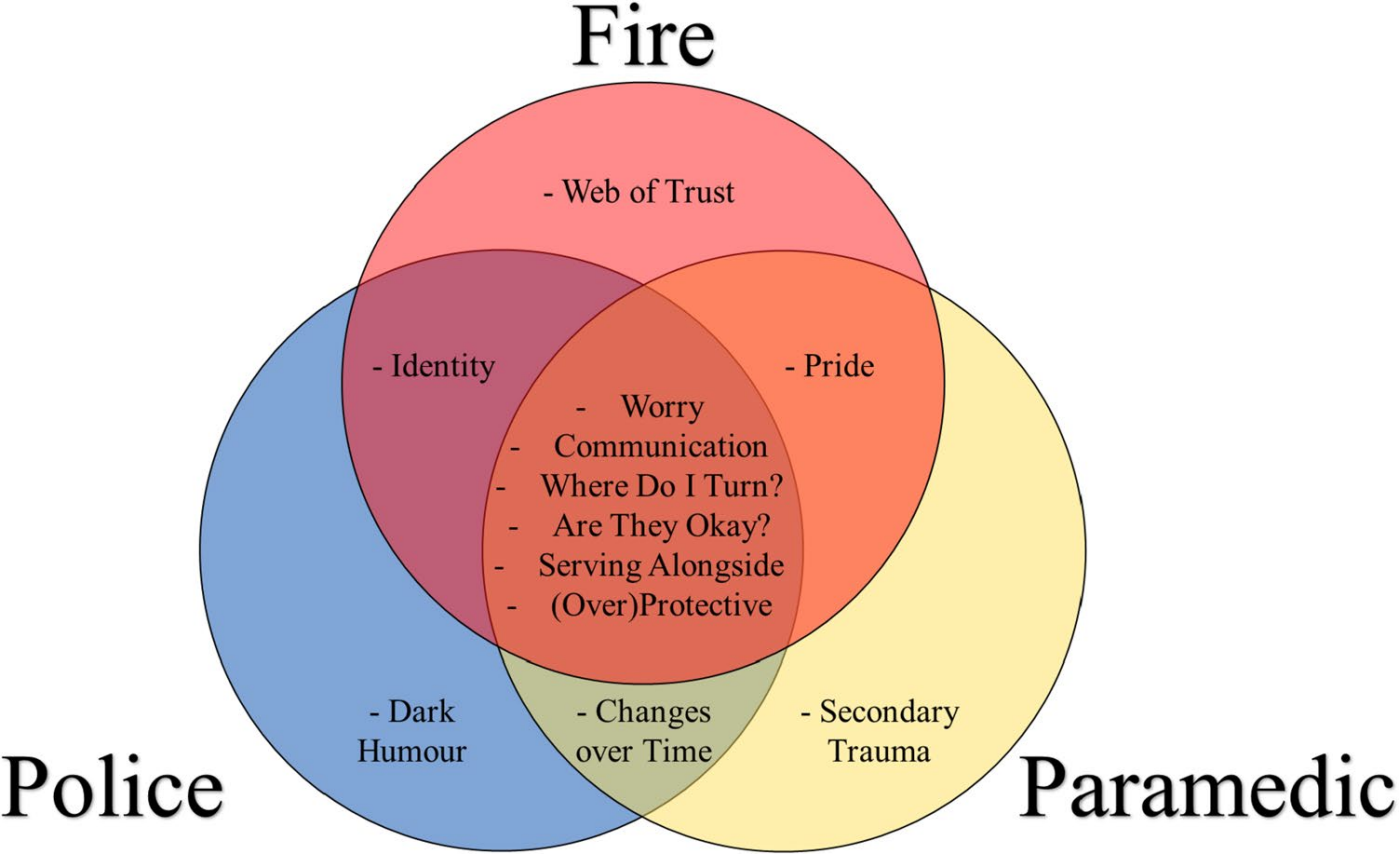
Themes by public safety sector

The 365 segments of unequivocal data from family members were divided and synthesized by occupational group: 71 firefighter segments, 103 paramedic, 169 police, and 22 segments from a mixture of those groups. All family members, regardless of occupational group, endorsed themes surrounding ‘worry’, ‘communication’, ‘where do I turn?’, ‘are they okay?’, ‘serving alongside’, and ‘(over) protective’. In addition, unique themes were identified within select occupational groups (see Fig. 1). First, we break down the shared themes by occupational groups, as well as the unique experiences of different sectors within those themes, and then we unpack the themes pertaining to specific occupational groups.

### Worry: Shared Experience

Families of firefighters, paramedics, and police expressed concerns about the safety of their loved ones at work given their occupational requirements, “I mean, it’s dangerous all the time … it’s still risky every day”—Police; [6]. The danger inherent to the occupation was a source of worry. This was particularly pronounced amongst families, without any discernible difference by gender of the PSP or family member, when their loved ones had previously been in danger or injured on duty: “My primary concern is somebody doing something to her. [So far it has been] mostly verbal, physically she’s been touched, an indecent touch now and then but nothing where she was actually punched, kicked, or anything”—Paramedic; [21].

In addition to demonstrated concern about the physical safety of their PSP loved ones, families disclosed trying to hide their daily worry and fear from their loved one, “I was beyond worried about him but didn’t want him to know”—Firefighter; [22]. Moreover, families described various strategies they implemented to reduce worry, while their PSP was on shift. “We would touch base every night before I went to bed … I just had to hear his voice … know it was okay and then pray that everything would be okay throughout the night”—Police; [6]).

#### Unique Experiences: Police

Beyond the worry families have about inherent dangers in the job, families of police expressed changing respect for police in society as potentially threatening: “Seeing the erosion of respect for police officers that has occurred over time—knowing that the uniform and the squad make him a target to some” [23]. The risk potential extended to officers’ home lives: “His squad has been egged, paint-balled, and had glass broken just sitting parked in front of our house” [23], which created fear. For instance, a family member was quoted in saying: “I worry that someone will want to get back at us … because my husband helped put them away or something. You hear things like that happening, people getting pictures of their kids mailed to them, that’s my biggest fear” [6].

#### Unique Experiences: Paramedics

Families of paramedics describe specific fears of their loved one being physically assaulted: “He’s knocked at the door and they met him with a knife or they’ve gone to a shooting and they’ve gone into a house and when they were checking the woman, the gun was under the bed” [24] or contracting an infectious illness: “2 months after we were married, he got a needle stick injury, he had to go for HIV testing … Then as soon as he came off that, within a month he got spat in the face and had to do the HIV testing again” [24].

### Are They Okay?: Shared Experience

Despite the type of PSP occupation of their loved one, family members described assessing and managing the emotional state of their loved one whenever they return home from work. “As soon as they come in the door, you try to read them. What their facial expression is telling you, their body language is telling you. Maybe even their tone. … Maybe he lost a baby. Maybe children were involved close to his children's age. You learn to read that and not take it personally”—Firefighter; [5]. In addition to spouses assessing the emotional well-being of their PSP, children also learned to read the PSP family member to determine if a shift was potentially difficult, “We’d pick the kids up from the sitter and my son would know that we had bad days just by the way we hugged them. I’d think—Ok this is too scary”—Paramedic; [24]. This reveals how interactions with children are affected by the strains of public safety work. Overtime, families indicated that they learned how their PSP acted and coped with the difficulties of work. Resultantly, one response was to ensure the PSP loved one had space to decompress after a shift:

The way I cope with it is I usually try to just stay out of his way. . . This is probably how our relationship has been for as long as we’ve been married. I don’t speak to him until he speaks to me, because that’s usually a sign that he really just needs to be by himself or just really disassociate himself from everybody until he’s ready to talk. And then I know its okay—Paramedic; [21].

This excerpt reveals how the realities of the job had lasting impacts on PSP, which required a distancing from loved ones to ground themselves, particularly after a trying day. Another response supported in the literature was for families to support their PSP loved one by trying to determine if their coping strategies were sufficient to manage the potential crisis from work, “He will do something and I’ll think, ‘I need to investigate it more to see what's going on there’”—Firefighter; [9]. As evidenced in the excerpt, PSP families worried about the quality and degree of support their loved ones received after potential psychologically traumatic event exposures, particularly when their loved one was outwardly showing the effects of their shift experiences.

### Communication: Shared Experience

Across PSP organizations, family members explained how they often encouraged their loved one to talk about their work day in the hope of providing support, “I am the safe person that he can say, yeah it completely sucked to see somebody run over by a train”—Police; [25]. Families, as evidenced in the excerpt, encouraged open communication, wanting and providing a space for their PSP loved one to speak to the events of their workday. However, they also noted how doing so could cause conflict in their relationship due to differing expectations of supports delivery:

As a young couple and as a bride, I thought he’d tell me everything and I’d be expected to help him with his way through that. I didn’t realize that he wasn’t willing to share that with me and I felt that I was failing as a wife. Now I think its important for him to keep the calls out of his home life and I’ve gotten to accept that as long as I know that there was something that happened, I can make sure that I take a step back and the kids take a step back and they don’t take any moodiness—Firefighter; [5].

Here, families felt “pushed away” despite desiring communication. They wanted to be present—a listening ear—for their loved one. But also felt there was a required degree of space that had to be offered to their PSP loved ones. For this reason, at least in part, families also understood why their loved one withheld information about work: “My wife does discuss some calls that she attends to, because I think it helps her to debrief, but I can see there are times when she wants to talk more about work, but may feel like it will burden me or cause me to worry about her”—Paramedic; [7]. As evinced in the excerpt, families felt their loved one tried to protect them–or was barred from communicating due to confidentiality requirements. Thus, in response, families needed to know if their loved one was speaking to anyone about their work experiences, ensuring they had some support:

When he comes home and I ask him if any thing’s wrong, he says ‘No.’ And then like a week later, he’ll tell his other buddies or he’ll tell other people and then I’m like, ‘I just asked you that like in a week ago. I’m here for you, and you’re telling everybody else’ who I think he feels more comfortable with, who are in the job or in a position that they’re in, and I’m not—Police; [26].

Families were keen to learn their loved one debriefed with someone about their work experiences. They understood that bottling up emotions was harmful overtime, thus simply desiring support for their PSP loved ones.

#### Unique Experiences: Firefighters

Some families did not want to hear about their firefighters’ shifts for their own personal coping. “He’s seen a lot of awful things and he will come home and start to tell me something and I, I just say, ‘Okay you have to stop that there now, have to stop because I can’t listen to that.’ … and he always says to me, ‘you just put your head in the sand’ … And I say, ‘Well that’s how I cope with things’” [27]. Thus, hearing was possibly tied to vicarious trauma and new degrees of strain.

#### Unique Experiences: Paramedics

The only literature presenting dual serving couples was that of paramedics. In these instances, spouses or significant others described having open communication regarding their work which actively improved their marital relationship due to the inherent understanding of the role and experiences arising from dual service: “We both work in the [medical] field and we thus talk to each other about incidents we may experience and this helps bring us closer, because as we understand what the other person may be feeling” [7]. Through a shared understanding of the work requirements, couples working in the same fields were able to communicate and debrief openly together.

#### Unique Experiences: Police

Families of police report that their loved ones withdraw emotionally and elect to discuss neither their emotions nor their work:

I feel that in order to maintain his own sanity and emotional well-being, my spouse detaches himself from his feelings. This causes difficulty between us because he has become essentially unable to get in touch with any emotion at all. I feel the negativity he sees at work on a daily basis causes him to mistrust others and have a negative outlook on life society—including family members [23].

Families notice their PSP withdrawing emotionally to cope with their work, and feel the impacts.

### Serving Alongside: Shared Experience

Families discussed making personal sacrifices, particularly on spending time together, to support the work of their PSP loved one. Supporting their work included ensuring their loved one could optimize their performance on duty (e.g. have enough sleep), “Sometimes I kid with him and I call myself the ‘EMS widow’ because that’s my sacrifice in understanding that he needs sleep”—Paramedic; [21]. Families of PSP described having to orbit around their loved one’s schedule and sacrifice their time. “It seems that our families are always trying to accommodate his schedule. I’m often embarrassed about it and feel guilty that others have to be flexible for it, but we can’t be flexible for them”—Police; [23].

Families describe their lives as going on without the PSP at all, leaving the spouses or significant others acting as single parents, responsible for child rearing, household responsibilities, and the limitation of their personal time, “We got used to not having him around. When the kids got out of school, he was already gone. He would come home in the morning and they were already gone to school”—Firefighter; [5]. As evidenced in the excerpt, loved ones of PSP report operating around the PSP schedule with the lion’s share of responsibilities being accomplished by the non-PSP family member in the absence of their PSP loved one. As a result, families must accommodate PSP schedules in a variety of ways but also adjust their behaviours while the PSP is at home: “They understand now that dad’s lying down and you have to be quiet. When they were littler that was definitely a challenge... and sometimes just going on with your normal day and trying to be quiet is hard”—Paramedic; [21]. Families emphasized the difficulties around ensuring their PSP has adequate sleep, especially with children in the home. The challenge, however, arose in how many families felt underrecognized for their sacrifices: “You train them, you spend a lot of money, and you need to make sure that you look after them. Because the other thing is, is that families, We deserve better than what they're giving us too, because we're picking up damaged people”—Police; [9]. These families of PSP loved ones described, through their sacrifices, serving alongside their PSP loved one. They enable the PSP to “do their work” by providing support across dimensions of personal living (e.g. socially, emotionally, applied).

While families want to support their PSP loved one, they did express resentment, with some feeling their needs were superseded by their loved one’s. “Sometimes there's a little bit of resentment that can be there… I’ll be like ‘I’ve had a bad day too.’ I always feel like my feelings sometimes would have to take the backseat to the fact that I don’t work in an industry where I'm saving lives and I'm not seeing trauma”—Firefighter; [28].

Being relegated to a secondary status on a proverbial hierarchy due to the implied necessity of public safety work for society, as per the excerpt, did cause harm. Family members felt unheard or trivialized, despite their challenges adapting the work of their loved one. Perhaps without surprise, some families felt their involvement in their partners’ lives resulted in some marital difficulties. “His inability to communicate and share emotions with me. It’s not difficult to be married to a police officer. It’s difficult being married to a person with the characteristics that make a good officer such as being controlling, and inability to show emotion and share their feelings”—Police; [23]. In this excerpt, the coldness and emotional detachment—a potential result of the occupational work—was pronounced and created marital strain. As echoed in the literature, marital strain resulted and was compounded by feeling secondary to their PSP loved one. This was exacerbated for family members of loved ones working in the police service if their partners had a posttraumatic stress injury: “It was awful. I felt like I was on eggshells all the time. He was irrational about everything, and it went on and on… I was saying, ‘I’m going to leave and go to another state and, you know, get away from this’”—Police; [27]. Families spent much time providing support for their PSP loved one, which took a toll on families in their care provision role and even their own wellness. This was echoed by others, who also felt PTSD was difficult to accommodate through a caring role and to help ensure triggers were avoided, “you're more aware of things that you have to avoid…”—Firefighter; [9]. To circumvent their PSP environmental triggers, families would also accommodate their commuting itineraries—avoiding locations where the PSP was exposed to traumatic events.

#### Unique Experiences: Paramedics

The constant need to conform to their paramedic’s schedule and not being able to rely on them at social events could lead some paramedic families to take up activities that did not involve their loved one and live with more flexibility, “I try new recipes that he doesn’t want to try, watch movies he doesn’t want to see, and I do sewing; I do crafts” [21]. Families would utilize their time away from their PSP to participate in desired activities that they felt unable to accomplish with their PSP at home. In addition, families of paramedics, perhaps amplified during times of large health crises (such as SARS), described being impacted by the work requirements tied to public health measures of their loved one, “Because he was quarantined, I didn’t go to work either… It affected my work because I didn’t want to freak everybody out at work” [24]. Though the public health measures may not have applied to the families of paramedics, they felt that it was their duty to sacrifice their plans to follow those measures.

Due to paramedics working closely through shared experiences with work partners, some loved ones felt their intimate relationship was under threat—creating apprehension and fears of intrusion into their personal relationship by a colleague, “Partners [of paramedics] are very, very close. Sometimes, perhaps from the spouse’s point of view, perhaps too close” [24]. Families felt uncertainty over the potential for intimate relationships to develop between partners at work given their shared experiences.

#### Unique Experiences: Police

Many families look at the difficulties they report in accommodating the PSP schedules and wonder what their lives would look like if their loved ones had chosen a different career path. “I mean there’s—with his accidents and all that there’s limits to our quality of time. It’s really been affected with the shift work and then with the accidents, because he’s not able to do many things and it’s the damage of the job that does it. You know he wouldn’t have had these things if he was an accountant” [25]. Constantly accommodating both the schedule and the consequences, as well as mental health symptoms associated with a high-risk occupation, families contemplate potential alternatives to their lives. The impacts were reported as intensified by how families, here of police officers, described the unhealthy coping behaviours of their PSP loved one caused problems in the home:

It’s actually really kind of like a giant tornado that runs right through our marriage when he goes out drinking with the guys after work to one of the cop bars. Honestly, I really cannot stand it anymore. Before I know it, one of those damn ‘copper hopper’ tramps is flirting with him and giving him their phone number. When I do his laundry and find these phone numbers, it's always a fight [29].

### Where Do I Turn (Peer and Organizational Support): Shared Experience

Families emphasized that it was crucial to have other public PSP families to talk to for support, as they understood the experience: “All of the spouses know what it’s like, we can go out together and they just really understand. We all understand the weird shifts, weird workdays, and it’s nice to have a support group, people that you identify with. It helps on the days when it’s hard”—Police; [6]. Being able to talk to other families of PSP, people with shared experiences who understand, as essentially the equivalent of having reliable and effective peer support for PSP families. This support is sometimes available through the PSP organizations, most families requested support for themselves from the organizations, “He got excellent support, but we got zero … nobody called me”—Paramedic; [24].

Despite the variation in if peer support was offered for families, families emphasized needing and wanting more involvement from the organizations, especially around support for the whole family. For example:

I would ask the fire department to get the families more involved. It’s not at all a family-oriented kind of workplace. You know, there’s no—I don’t think X has any family-related leave or—You know what I mean? There’s no sort of focus on family—But I think a lot of wives don’t have any idea of the shit these guys see. Sometimes I just—I think it would be nice if the management engaged the families a bit more—Firefighter; [30].

As this excerpt demonstrates, families are asking to be more involved and supported by the PSP organizations, to be included in the comradery and to share in providing support for themselves and their PSP loved ones.

### (Over)Protective of the Family and Surroundings: Shared Experience

Families of PSP expressed how their loved ones often remain hypervigilant and protective of them and their communities after leaving work. They had a more intensive understanding of what constituted safety, less trust in the public, and, in consequence, carried the additional weight of ensuring their families safety and wellness. “I can’t even go to Wal-Mart past dark anymore! Not by myself anyways. If I go past six or seven o’clock, he has to come with me and his head is always on a swivel. He’s so protective now that he knows all the bad stuff that goes on. I understand it, but it’s still frustrating”—Police; [6]. Families described extra ‘safety’ precautions employed by their PSP loved ones. However, while families understand the level of protection offered and tended to, the hypervigilance was also a contribution to feelings of embarrassment: “If he sees smoke, we have to follow it. I’m sure we look ridiculous going up to someone’s house just to see if they have their burn under control. I just hide my face and pray I don’t know them”—Firefighter; [6]. Families understand why their PSP feel the need to remain alert and assert safety in the home–due to their experiences at work–yet families remained uncomfortable and sometimes humiliated by the imposed restrictions, feeling their own agency and freedom was impinged upon.

### Identity: Shared Experience of Firefighter and Police Families

Families of both police and firefighting sectors explained that the public safety service was as much a part of their identity as it was for their employed loved one. “You absolutely get engulfed … you sort of get sucked in and become part of it if you see what I mean? It’s … more than just a sort of job thing it’s a way of life thing”—Firefighter; [31]. “To serve and protect is not just a motto, it’s a saying that we live by”—Police; [6]. While identity is ingrained in both police and firefighting families, as evidenced in the excerpts, the impacts of that imposed identity differ across services.

#### Unique Experiences: Firefighters

Families of firefighters reported positively associating with the firefighter identity and encouraging not only their children but others in the community to join. “If someone said, ‘I know this guy and he’s a police officer and I know this guy who’s a firefighter.’ I’d say ‘Marry the firefighter’ or if some girl says ‘I want to marry this police officer’ and I say ‘Find a firefighter’” [5]. Here, perhaps counter to other PSP identities, the perception around firefighting was extremely positive for the family as a whole, as well around the community.

#### Unique Experiences: Police

The changing public interpretation of the police had negative affects on families—who felt the impacts of the scrutiny directed at their PSP loved one and even themselves due to their association with a law enforcement agency. Police families specifically conveyed their association with the agency more negatively, explaining they have to comport themselves differently due to their association: “Having to live by a higher standard than family/friends live by, which may cause conflicts within these relationships” [23]. To offset many of the societal perceptions, police families feel the need to behave to a ‘higher standard’ than their peers, which is both exhausting and can cause rifts amongst those relationships.

Changing public perceptions also results in the children of the police to have to reconcile the societal perception that all police are ‘racist’ and ‘bad’ with the fact that their parents are good, safe, and trusting members of the police service:

A lot of time people are saying the police are too rough or killing people for no reason. But what I have seen is they are not doing anything too wrong they tell people to stop multiple times. They are just hating on the police and not doing any justice and making the world more wrong. It just bugs me because I know that police are there to help out and the more you push the cops away the more trouble you will get in. […] I know that every cop is trying to help and under pressure they are to deal with it. It’s unfair what is said. People really don’t know what the issue really is [32].

The children of police, as evidenced in the excerpt, need to navigate the misalignment between how they understand their loved one’s work versus how the public perceives the work of heir loved one. In light of this, police family members discourage the police lifestyle heavily for others as well as their own children, “My guidance counselor mentioned it because we do career tests, but when I mentioned it. My dad said he didn’t want me to do that. There are things happening to police right now and he said it is not a good career choice” [32]. Despite potentially wanting to follow in their police parent’s footsteps, children are discouraged of entertaining this career path due to the change in societal perception of the police.

### Pride: Shared Experience of Firefighter and Paramedic Families

Families of paramedics and firefighters emphasized being proud of their loved one’s work, “Being a part of the fire community is like being, you know, a part of something bigger. My husband does a dangerous job, but he also gets to interact with the community in different ways, and that is the cool part of the job”—Firefighter; [6]. In addition to the pride and positive impacts that families feel towards their loved one’s work, the work continued to have a positive impact on their family as a whole, “It has a positive impact because of the way she helps people and she also brings that caring nature into our relationship and helps me as a person”—Paramedic; [7]. Families of both paramedics and firefighters are honoured by the work their loved one’s do both for the community and in the home.

### Changing Over Time: Shared Experience of Paramedic and Police Families

Families of police and paramedics explained how they witnessed the behaviours of their loved ones’ change overtime as they worked in their public safety sector, for better and for worse, “The job is never over; it’s a part of who he is as a person. Being an officer has changed my spouse. He is not as joyful and carefree as he once was in college. He trusts no one”—Police; [23]. Over time, families noticed how their PSP loved ones became less “carefree” and “harder”, increasingly affected by the hardship that comes with the job. The hardness was also a response to psychological trauma–a boundary to help cope with the realities of their work and all that they work exposed them too. These changes were not only noticed by family members but also directly impacted their family members. “If I was to tell him I was sick, before he was a paramedic he used to hug me and cuddle me. Now he’s like ‘suck it up.’”—Paramedic; [5].

The learned toughness and overall changing of character expressed by PSP result in feelings of sadness and loss amongst family members—a sort of mourning for who one once was. While families mostly remarked on the negative changes their loved ones experienced over time, they also noticed positive coping mechanisms that developed: “She learned to deal with [it]. She’s found her way. Early on she came home really upset. Other times she’d come home and not talk for a week or so. She’s definitely changed and matured in the way she deals with [it]. She found her zone, I guess you could say”—Paramedic; [5]. These positives appeared to be held tightly to a counter influence to the negative changes families noticed in their PSP over time. The positive coping expanded to help make managing the intricacies of daily living and keeping realities in perspectives. Basically, families celebrated the development of healthy coping mechanisms and implementing healthy strategies in response to challenges.

### Web of Trust—Firefighters

Firefighter families emphasized trusting the judgement, training, decision-making skills, support, and equipment their firefighters draw on to keep them safe while out on calls, “I think wherever possible people are trained for every eventuality and they’re wrapped up in cotton wool with health and safety and risk assessments and everything else, really got to be something fairly unexpected and significant for a firefighter to be injured” [31]. The comradery of firefighters was keenly welcomed by families, who trusted in their firefighter loved one and their colleagues as well as their training, equipment, and the support received from their organization and fellow firefighters. This provided some needed reassurance about the ability to stay safe of their loved one on the job.

### Secondary Trauma—Paramedics

Vicarious or secondary trauma was noted in the literature on paramedics. Here, families want to be supportive and help their paramedic decompress, but also feel impacted by discussions tied to the realities their loved ones experience daily when completing their shift:

Depending on the incident, I can feel traumatised myself, but sometimes I feel really sad for the patient, or really upset if a child is involved in any way. He doesn’t often talk about the death of a patient, but if he does, it can be hard to hear and make me worry about his wellbeing. I also tend to then think of my own death or if something had to happen to him. [7].

Those spouses and loved ones who had not worked in the healthcare industry expressed having nightmares and other posttraumatic symptoms relating to stories their paramedic shared with them—clearly impacted by the vicarious trauma.

### Humour—Police

Police also often use humour as coping mechanisms and to share the lighter aspects of their work with family, “If he finds something funny that happened at work or something that he thinks he did that was funny, he would definitely share it” [26]. While this use of humour has mixed results amongst family members, families also use humour to mask their fear towards their police loved one as well:

I couldn’t run away from him or ever tell him how scared I was that this could happen to him again and force him to quit. Policing is his life and I couldn’t take that away from him so I pretended I was okay. I even cracked jokes telling him that at least his video game hand wasn’t affected by the shooting. We both laughed. [22].

Humour, as evidenced in the excerpts, is employed both by the police and their families when communicating aspects of the job. Humour, including dark humour, is used as a coping mechanisms to hide worry and manage the harms of which they are forced to become aware—a counter to the darkness of social living that becomes front and centre when their loved one’s job is to respond to situations of crisis or transgressions of the law.

### Family Experiences by Familial Role

The 365 segments of unequivocal data were also divided and synthesized by familial role and gender, where possible, yielding 236 female spouse’s or significant other’s segments, 32 male spouse’s or significant other’s segments, 36 segments from spouse’s or significant other’s where no gender was indicated, 56 segments from children, 4 segments from a mother, and 1 segment from a brother of a PSP.

### Children

The data from children were primarily from children of police, despite originating from three articles [22, 29, 32]. These children ranging from 10 years old to adult children spoke mainly about: worry, let’s laugh instead, (over)protective, and I’m not the police, my parent is.

#### Worry

Overall, children worrying for their PSP parents’ safety does not differ drastically from those of other family members, “Just the overall worrying about his safety. […] It is just not knowing what is out there. Not like my friends’ parents that work a regular job. Police officers are just different because of the hours they work and what could happen” [32]. Children express worry in terms of the uncertainty, the danger, and the feeling of injustice they experience directed towards their parent’s wellness.

For some children, they rationalize based on the kind of work their parents do that they will be safer than others. “Luckily, my parents don’t work patrol officer duty. It is those officers that can get hurt the worst. Then it just gets worse for those in bigger cities. Even though my parents don’t work in a real big city, I still worry about all officers. They are heroes doing an honorable job” [32]. Unfortunately, the reverse is also true, where children were very aware of the precise risks that their parents’ current position held, “My mom works the night shift and that is when more crazy people are out there—just people doing stupid shit. It is scary because I know she can get hurt” Helfers et al. [32] p. 245. Children of police also described the added worry they feel due to the changing public perceptions of police—particularly in light of dialogues about police militarization, Black Lives Matter, and increased police shottings. “The ambushes we have heard about is really scary. You just have cops out there minding their own business and get shot just because they are cops” [32]. Children are very aware of the potential harms their parents face while at work and worry prevails, similarly to the experience of the rest of their families.

#### Let’s Laugh Instead

Children also spoke about humour in a few ways. The first referred to disliking their parents’ use of dark humour at home, “When my friend died in a car accident during final examination week, my dad joked that at least she didn’t have to worry about passing her exams! I didn’t speak to him for months” [22]. Dark humour, as apparent in the literature, was not always well received by loved ones and could at times cause rifts in the familial relationships. Police parents would also use humour to share stories from work in a non-frightening manner to reassure the children about the dangers of their work and as an educational tool to discourage bad behaviour; however, these stories did not always have their intended effect, “My dad might think those funny stories help but I know he is hiding the bad stuff from me. I’m scared everyday about what he does” [29]. This type of storied humour often made children wonder what more dangerous scenarios their parents were experiencing at work–what was not being disclosed–which provoked more worry amongst children. Children also spoke about how dark humour was a coping mechanism to hide their fear from their parent in hopes that, if the parent were unaware that the child was scared, they would be better able to perform the tasks of their job. “What good is it for my dad to worry that I’m worrying about him? If he knew that, he wouldn’t be in the right mindset and I need him to come home after his shift. I’m all smiles when he leaves for work and I always ask him to share funny stories so we can laugh” [22]. In a similar fashion to the rest of the family, children utilized humour to mask their worry from their policing parent.

#### (Over)Protective

Children also spoke about how their lives are affected by their parents’ police occupation because of their resultant hypervigilance and need to protect the safety of their child and families. Hypervigilance was perceived and described in a few ways. For instance, some children felt that the imposed protection of their police parents was positive, leading to them feeling knowledgeable and safe, “I just feel safer because I think I know more ways to be aware of what is around me. My friends’ parents just don’t know. I think he also keeps me safe by asking a lot of questions” [32]. This form of protection was received positively, associating the behaviours with their enhanced safety and knowledge of the law. Other children reported understanding why their parents were more protective over them due to their professions, but also did not appreciate the heightened restrictions and protectiveness that resulted. “I just get watched over way more than my friends do. That isn’t something I like. I understand why my mom does it, but I don’t like it. I just don’t have as much freedom” [32]. Despite being aware that their parents’ overprotective behaviours originated from a place of knowledge and safety due to their work, children did not always respond positively. Lastly, children described being embarrassed by their overprotective parents restricting their freedom, which they equated with being excluded from certain social experiences. “I hate that my mom is a police officer because I can't do anything. It's like a battleground in my house when all I want to do is go to the mall with friends. She has to tell me stories about kidnapping and child abductions that happen at the mall and then only lets me go if she can escort me. It's embarrassing!” [29]. This form of overprotective behaviour was viewed negatively by children, particularly when the overprotectiveness resulted in not being able to participate in group activities, which led to resentment towards their policing parent who were considered strict.

#### I’m not the Police, my Parent is!

Children described being blamed for negative actions that police have taken as if the children were police officers by association, “I have been made fun of because this one kid’s mom got arrested. Like I was the one to blame? I didn’t do anything, but I have been picked on because the police are just out there doing their job” [32]. Despite not being members of the police service themselves, children of police feel treated as though they too represent the service and greater governing body. Children explained how people expect them to know and have opinions on legal and police-related matters, despite them only being children and outside the profession. “I kind of get used as a resource. So when something comes up they ask me what I think. […] You know they think that since my dad is a cop I know what he does or what the law or how things happen. I ain’t a cop and I ain’t a lawyer” [32]. These children felt unjustly used, with their peers expecting them to have the expertise of their parents despite not having the job.

Children also reported being bullied and harassed by their peers for no other reason than their parents’ occupations:

Some kids think just because my mom is a police officer that I automatically am going to run to her and tell her about something they are doing. They call me a snitch when I have never told my mom anything about their parties. They just assume. I could care less what they do, but I am not going to hang myself out there by ratting on them. It’s a nuisance, like being harassed [32].

Children of police felt they were treated differently than their peers, with their peers assuming that due to their parents’ professions they will act like them and report any misconduct to the authorities. This assumption of behaviours is worsened with the changing societal and political climate towards the police. “I get teased and told that I’m a “goody goody”. I’ve been called a racist because my dad is a cop and cops are harassing Mexicans. My dad doesn’t work at the border. He just does his job. It’s just annoying when I hear comments like that” [32]. As evidenced in the excerpt, children of police have been harassed due to the interpretations of the actions of police despite those actions not representing themselves or even their parent.

### Spouses and Significant Others

Spouses and significant others did not differ in their descriptions of their experiences based on identifying as a male or female. The descriptions were also similar regardless of the identified gender of the PSP. All spouses or significant others discussed issues around shift work, communication, psychological trauma, and mental health, coping mechanisms for both themselves, and the PSP, safety, and children when applicable. The only notable difference in descriptions occurred if a couple was dual or single serving, especially surrounding communication.

#### Single-Serving Household

When a family was composed of a PSP and a civilian, descriptions fell under three main categories. The first category is that the PSP is withdrawn and does not communicate about the nuances of their work to their family. “It impacts our relationship because there are times when he pushes me away and becomes distant, and this upsets me because I like to have open communication and to be able to support him” [7]. In these instances, the families desired open communication, and the PSP chose to remain closed off, resulting in negative relationship consequences. The second category involved the PSP communicating very little about the details of their work, trying to balance keeping open communication and not frightening or worrying their spouse or significant other further:

I think sometimes he comes home with an attitude or like a shield and I think that sometimes it gets hard, ya know to deal with that, because I’d like to know what’s happening, but at the same time I don’t. So it’s hard. But then if he just, you know, if I say did you have a bad day or did something happen and it’s just, ‘Yeah it was just not a good day’. And I just kind of know the fact that something happened and I don’t want to know anyway [25].

In this case, the families understand why the PSP is withholding information and they both appreciate the gesture while also worrying about what the PSP is withholding. Lastly, the PSP communicates too much about work and leads to the family experiencing secondary psychologically traumatic stress symptoms, “It does affect me if he describes an incident that involves children, like a young child in a car accident and the child has passed away. He told a really terrible story about a patient that was a child and I cried when he told me” [7]. In these situations, the PSP communicates openly about the details of their work; however, the family member is not prepared to hear that information and can result in them experiencing negative mental health consequences.

Another component that was different from single serving to dual serving households was the level of advocacy. Single serving families expressed that they knew their PSP had access to resources through the organizations, but that they chose not to use them, “My husband feels it is not safe to seek help with any mental health issues that may arise as this information is not kept confidential” [33]. While families acknowledge that services and resources are available to their PSP, they call for the organization to make improvements to ensure their PSP actually feel comfortable using them properly. Similarly, these families also call for the organizations to implement programmes or resources for themselves:

And I just felt like there’s nowhere to go with this stuff as a, as a parent who has been dealing with this issue with her for 2 years… I’ll probably start crying, but the other week I was so bloody stressed that I felt like I couldn’t breathe...who is there to contact as, as a supporter of a responder when you’re also dealing with the difficulties of what they’re dealing with, where do you go? What do you do? All I want is a phone number of somebody who if there was some system for families…to be able to contact a social worker or speak to somebody and say, ‘Look things are not going well, can you check on this situation? Can you follow through’ [27].

While the resources may exist but are not utilized by PSP, the families are actively wanting and seeking these supports for themselves.

#### Dual Serving Households

Dual serving couples, on the other hand, primarily from the paramedic sector in the literature, described having healthy open communication stating that their spouse or significant other would understand and support them, “I think, had she been someone else probably, I wouldn’t, but I know that she’s been there, and she’s seen it, and it’s kind of a way to decompress a little bit just to get it out” [26]. Dual serving families felt comfortable debriefing about their workday with their SSO, knowing they would not only understand but would not be triggered in the same way as a non-PSP. These families explained that, while they understand the dangers and challenges of their partners’ work, they have less concerns and fear than single serving families, “I do [worry], but being that we are both knowledgeable about [risks], it is not as much of a concern” [21]. Knowing the realities of PSP work, lessens the worry dual serving couples have towards their loved ones’ work.

Similarly to single serving families, dual serving families also sought support for their PSP; however, many of them went outside of their organizations due to their knowledge of the systemic difficulties associated with accessing and using available supports:

Because the culture has always been, teaspoon of cement. Suck it up, go to the next job. And also, there’s that stigma attached to going to health and welfare. You know, so, there’s psychs in there that you can go and see, but you don’t, because you don’t want to ruin your career. You feel weak if you go in and say that there’s a problem [27].

Dual serving families seek supports outside of the organization both for themselves and their spouse or significant other after having poor experiences with their own organization’s supports. These families also expressed some disparities in how they view these situations, given the diverse roles they hold–that of being a fellow PSP as well as a family member. “Commanding officers are brilliant… But like as a partner I’d be like, no way in hell, because now I’ve dobbed on my partner to the same organisation that he does not want help from. How dare I, and now I’m in the shit again” [27]. Dual serving couples added another level of complexity for communication, as evidenced in the excerpt, in which they may need to decide where their duties lie, either to disclose with the organization in their occupational role, or with their family and protecting and supporting them in that capacity.

## Discussion

This review sought to identify and describe the experiences of families of PSP as they navigate through the occupational demands required by the profession and public that are inherent to public safety work. We found shared and unique experiences for families in available literature for families of paramedics, firefighters, and police, but found a gap in the knowledge around the families of other PSP, such as correctional service providers and public safety communicators. Thus, there clearly remains a need for more research in the area. We also want to contextualize our results in another limitation, simply because a theme was found as unique to one public safety profession, this could be a result of the limited research in a given area of public safety families. That being said, available research supports that, just as there are similarities in occupational risk across military and public safety sectors, so too are there shared experiences for their families. Military families face specific lifestyle factors and challenges such as relocation, parental absence, and risk of illness or injury [34, 35]. These factors, in addition to the occupational stress that the member brings home, impact family well-being [34, 36, 37].

Public safety families also face comparable challenges and dimensions in relation to the occupational factors that shape family lifestyle and incite stress spillover [5, 24, 38]. Moreover, there are many shared experiences amongst the families of different types of PSP [39–41]. We also found sector-specific experiences in the literature; however, they are limited in the knowledge around how unique those experiences are. Our findings evidence a need for researchers, employers, stakeholders, and policy directors to consider and learn more about the families of PSP, regarding both their shared experiences and any experiences specific to their sectors.

Our review revealed that families of the three represented public safety sectors worry for the safety and well-being of their PSP loved one, physically, emotionally, socially, and psychologically. Our findings are consistent with both quantitative research in this area, as well as amongst both career and volunteer PSP [40–44], as well as families employed in similar high-risk occupations (e.g. military) [34]. Thus, the risk tied to these professions is a consistent worry for families. The risk inherent to the job weighs on PSP as well as their loved ones.

Our review identified organizational stressors across police, firefighters, and paramedics, which impacted families that may be remedied with organizational change. Families expressed difficulties adapting to the schedule of PSP shift work and determining who will maintain the household and child rearing responsibilities, as well as making personal sacrifices for the sake of their loved one’s career, even feeling the career of their loved one was prioritized in their family scheduling. While these findings were consistent amongst PSP, these experiences are not exclusive to families of PSP [45, 46]. Efforts are perhaps necessary to learn about different feasible schedules and what works in terms of shifts for PSP. Research supports that non-standard work, referring to those operating outside of the constructs of standard employment such as performing shift work at varying times, is burdensome [37, 38, 44–48].

Families of PSP also expressed strong yet differing experiences towards intrafamilial communication. Single serving families reported wanting to support their PSP and ensure that they shared their emotions and psychological trauma to help them process each and maintain their wellness. A possible outcome of this type of sharing is that it can result in secondary traumatization for the family member. Thus, there is evidence to support that some family members of loved one PSPs may require psychological supports (e.g. peer support, counselling) as well. This also includes in the realm of marital or family counselling given our findings suggest working in public safety can cause marital strain and the strain can come from different sources (e.g. secondary/vicarious trauma, relationships with close co-workers, schedules). Dual serving families, however, described having open communication regarding occupational risks and requirements, including reporting to value opportunities to discuss the events of their shift. This communication also could function as a coping mechanism or support system, thus suggesting dual serving families benefit from an expanded network of informal, and likely formal, supports and resources (see also [49]).

Families of PSP described how having occupationally informed supports were crucial to them feeling understood, and thus their personal coping and wellness. Both military families and health care providers to these families emphasized the uniqueness of the lifestyles, necessitating competencies when providing and receiving effective support [50, 51]. Peer support appears to function latently to help normalize one’s experiences, despite the manifest function of providing aide and advice based in evidence and experience [52]. The support of other PSP families also can help manage the stigma some families experience. For police, this stigma comes from the negatives in public perception of law enforcement. For all, the stigma around mental health more generally remains a barrier to treatment seeking [53]. It is also crucial to note that families across sectors emphasized the importance of knowing that supports existed for their PSP despite the stigma and likely lack of use. This is consistent even with police recruits demonstrating overall high stigma and low intention to access services, although these improved with more knowledge of mental health [54].

Our review demonstrated that some PSP families shared experiencing their PSP loved one as being hypervigilant and protective. Despite understanding why, many explained feeling embarrassed at times and even resentful of this behaviour. This behaviour may also have impacts; for instance, a study by Pereira, Barros [55] found overprotective parenting behaviours and concern were associated with a child experiencing anxiety. This was mirrored in military families, where parental overprotection was found to impair children’s interpersonal relationships and hinder adaptation to early adulthood [56].

In this review it was noted that, while many experiences of public safety families are shared amongst PSP sectors, there may be nuances in each sector. It is important to note that while this review notes the nuances as presented from the literature, it is possible that those nuances are apparent in other sectors but are limited in the literature. This too was echoed in military families, where there are many overarching similarities. However, each branch of the military may experience them slightly differently; for example, while military families as a whole experience relocation, the frequency differs due to a variety of factors such as service branch, specialty, etc. [57, 58]. Some of these nuances are evident in how families experienced worry both for their PSP loved one and the position as a PSP family member. Specifically, all families shared a worry for their PSP’s physical safety.

Firefighter families really emphasized having pride in their loved ones’ work and embraced the positive identity that being part of a fire family had on them, both at home and in society [5]. Paramedic and police families did not describe the same positivity, worrying more that their loved one’s harm potentiality could follow them home and potentially cause dangers to them in different ways, specifically for paramedics through the passing of communicable diseases [21, 24]. Police families particularly worried because of the diverse public perceptions of police [25]. This also impacted identities of PSP families, where firefighters’ families embraced being part of the firefighter profession, but police families did not as often [32]. Another difference evidencing diversity in worry is how families of firefighters expressed more pride towards their children becoming firefighters, even suggesting people should want to marry into firefighting families [24]. Police families, on the other hand, especially in recent years, are more likely to strongly discourage their kin and others of following their policing career paths, and even of dating or marrying into the profession [32]. While these experiences fall under the same categories, the nuances exhibited by each sector are crucial when considering developing programmes and policies for these families.

## Limitations

Our review is not without limitations. The first limitation being we may have inadvertently missed relevant literature. However, to mitigate this limitation, we utilized the expertise of a librarian, implementing targeting search terms, and cast a wide net with extensive screening procedures. These steps were put in place to ensure, to the best of our abilities, that all the relevant data would be identified. More relevant is that the existent literature appears to be limited both by presence and scope. While literature is available regarding these families, it is often from the PSP perspective and quantitative in nature, inadequately representing the proper lifestyle experiences of these families. Additionally, this review is only able to present upon published findings from the literature which is limited by the studies scope as well as the authors potential biases. To mitigate this and not re-iterate and re-introduce the former authors bias, we used the JBI methodology as well as meta-aggregation, both of which are well known and established manners in which to conduct and analyse qualitative systematic reviews in a scientific and rigorous fashion. It is also important to note that the experiences of these families differed during and after the COVID-19 pandemic, which are not fully captured in this review.

A concept that was not highlighted in this review, but is faced by families of high-risk professions, (for example, PSP, military, nursing) was the difference in experiences across the life course [59–61]. While this review found mentions of experiences from different stages throughout the life course, whether it be early, middle, late or retired in the career trajectory, or from different stages of the family life course, there were not enough excerpts to properly analyse across the life course [5, 21, 27]. The experience of a young member of the military with a new spouse and baby getting ready for deployment differs drastically from that of high-ranking member of the military that no longer deploys with grown adult children, and from dual serving middle-ranked members with no children [61]. Each stage of career and different familial compositions bring their own challenges and experiences and will require different supports [59–61]. While this concept has not been studied in depth amongst PSP, future research should consider understanding these familial experiences across the life course.

## Conclusion

The occupational risks and requirements of PSP transfer into the home and impact families. Through identifying and synthesizing these experiences of ‘Worry’, ‘Communication’, ‘Where do I turn’, ‘Are they okay’, ‘Serving alongside’, and ‘(Over)Protective’, across public safety sectors and by familial role, this review fills a gap by further understanding these families. Our review demonstrates where familial experiences overlap across public safety sectors and where they appear to differ. Unequivocally, families see themselves as inextricably linked with the PSP occupation and associated risks and requirements. These risks and requirements can take precedence over other forces shaping family life; our review has given shape to the shared phenomenon of PSP family life. In so doing, we have exposed the unique experiences of individual family members as shared patterns by which families are impacted.

This review named and framed the occupational forces that impact families. This both validates families’ experiences and externalizes them as forces beyond their own creation, thereby creating novel opportunities for prevention and intervention. Families have experiences and needs, in their own right, associated with their PSP loved one’s job; recognizing and responding to those direct and indirect needs should be a priority for PSP organizations. As we found that families support their PSP and help ensure their occupational readiness, it is crucial to ensure that the family is well supported. This review also underscores the need to support families in a preventive vein in managing the difficult balance of dynamic and diffuse challenges related to logistics and identities, ahead of any risks that may be incurred through the career life course. Keeping families well, physically, psychologically, and emotionally, may reduce the work-family conflict inherent in these occupations and support organizational recruitment and retention efforts.

## Supplementary Information

Below is the link to the electronic supplementary material.

Supplementary file1 (PDF 279 KB)

## Data Availability

The datasets used and/or analysed during the current study are available from the corresponding author on reasonable request.
