# Supplementary material for: Exploring the Occupational Lifestyle Experiences of the Families of Public Safety Personnel
Source: J Occup Rehabil. 2024 Mar 28;34(4):895–912. doi: 10.1007/s10926-024-10179-x (PMC11550274; doi:10.1007/s10926-024-10179-x)
Supplement: Supplementary file 1 — Supplementary file1 (PDF 279 KB) [file 10926_2024_10179_MOESM1_ESM.pdf]

Appendix A

Specific search strategy used in Embase (Embase Classic + Embase) is shown below. These search strings were modified as appropriate with the aid of a librarian for the other included databases.

Search	Query	Records retrieved
1	public safety personnel.mp.	45
2	emergency personnel.mp.	458
3	emergency respon*.mp.	4,470
4	blue light*.mp.	11,451
5	detective*.mp.	2,130
6	constable*.mp.	126
7	deputy chie*.mp.	58
8	superintendent*.mp.	658
9	sergeant*.mp.	277
10	uniformed officer*.mp.	4
11	platoon*.mp.	208
12	auxiliary.mp.	32,067
13	rcmp.mp.	125
14	red serge.mp.	0
15	mountie*.mp.	2
16	federal police.mp.	103

17	northwest mounted police.mp.	0
18	r c m p.mp.	1
19	royal canadian mounted police.mp.	58
20	police.mp.	22,932
21	tactical population*.mp.	9
22	fire inspector*.mp.	4
23	fire code*.mp.	18
24	(fire and rescue*).mp. [mp=title, abstract, heading word, drug trade name, original title, device manufacturer, drug manufacturer, device trade name, keyword, floating subheading word, candidate term word]	1,281
25	marine rescue*.mp.	2
26	trench rescue*.mp.	1
27	high angle rescue*.mp.	0
28	hazmat.mp.	231
29	hazardous material*.mp.	1,625
30	international association of fire fighters.mp.	6
31	coast guard*.mp.	313
32	ambulance*.mp.	21,000
33	base hospital*.mp.	923
34	paramed*.mp.	34,408

35	emergency medical responder*.mp.	34
36	emergency medical service*.mp.	14,252
37	emergency medical technician*.mp.	1,489
38	ornge.mp.	10
39	air ambulance*.mp.	814
40	paramedic*.mp.	29,145
41	volunteer emergency service*.mp.	3
42	community first responder*.mp.	39
43	call taker*.mp.	84
44	dispatcher*.mp.	1,114
45	communications officer*.mp.	12
46	security classification.mp.	13
47	detention cent*.mp.	720
48	correctional cent*.mp.	138
49	penetentiar*.mp.	3
50	remand*.mp.	530
51	inmate*.mp.	6,719
52	parole*.mp.	1,498
53	statutory release*.mp.	1
54	probation*.mp.	2,387
55	case manager*.mp.	5,040

56	caseworker*.mp.	409
57	incarcerat*.mp.	15,877
58	doing time.mp.	32
59	correctional service*.mp.	174
60	program officer*.mp.	81
61	forensic service worker*.mp.	0
62	forensic nurs*.mp.	735
63	prison staff.mp.	286
64	correction* officer*.mp.	213
65	rescue personnel/	7,759
66	halfway house/	1,461
67	police/ or emergency police dispatcher/	13,204
68	fire fighter/	3,149
69	prisoner/	17,814
70	or/1-69	189,557
71	family life/ or family coping/ or family functioning/ or family interaction/	16,312
72	family stress/	2,321
73	family/ or adopted child/ or adoption/ or adult child/ or childlessness/ or dysfunctional family/ or family decision	138,170

	making/ or family life/ or family relation/ or family separation/ or parenthood/ or single-parent family/	
74	spouse/ or domestic partner/ or husband/ or wife/	19,633
75	significant other*.mp.	5,331
76	exp marriage/	73,187
77	couple*.mp.	437,379
78	co habit*.mp.	399
79	cohabit*.mp.	6,670
80	or/71-79	661,445
81	70 and 80	4,792
82	limit 81 to (article or article in press or "review")	3,808

Note: .mp stands for multi-purpose, * indicates that all possible terminations of the term will be included in the search, rcmp refers to the Royal Canadian Mounted Police, Mountie is a colloquially used term for the Royal Canadian Mounted Police, Ornge is a Canadian not-for-profit corporation and registered charity that provides air ambulance and associated ground transportation services for the province of Ontario, under the direction of the province's Ministry of Health.

Appendix B

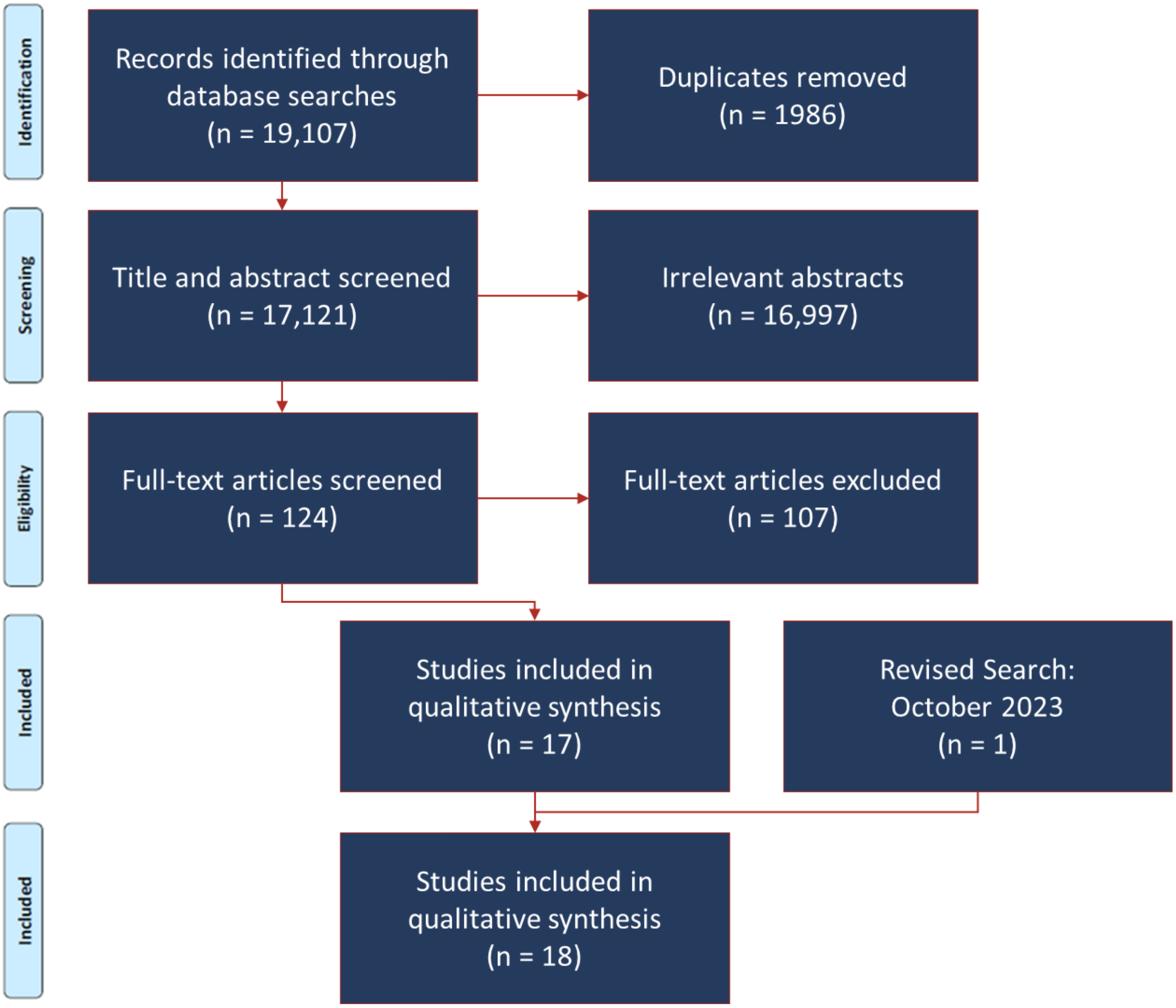
Identification
Records identified through database searches (n = 19,107)
Duplicates removed (n = 1986)
Screening
Title and abstract screened (n = 17,121)
Irrelevant abstracts (n = 16,997)
Eligibility
Full-text articles screened (n = 124)
Full-text articles excluded (n = 107)
Included
Studies included in qualitative synthesis (n = 17)
Revised Search: October 2023 (n = 1)
Included
Studies included in qualitative synthesis (n = 18)
